# Optimal Design Methodology of Tapered Waveguide Transducers for Thickness Monitoring

**DOI:** 10.3390/s20071892

**Published:** 2020-03-29

**Authors:** Jiuhong Jia, Yue Ren, Weiming Wang, Zuoyu Liao, Xiancheng Zhang, Shan-Tung Tu

**Affiliations:** Key Laboratory of Pressure Systems and Safety, Ministry of Education, East China University of Science and Technology, Shanghai 200237, China; yuerenae@163.com (Y.R.); wmwang_1@163.com (W.W.); zyliao@mail.ecust.edu.cn (Z.L.); xczhang@ecust.edu.cn (X.Z.); sttu@ecust.edu.cn (S.-T.T.)

**Keywords:** harsh environment, structural health monitoring, tapered waveguide unit, the quasi-fundamental shear horizontal wave, optimal design

## Abstract

For the purpose of providing transducers for long-term monitoring of wall thinning of critical pressure equipment in corrosion or high temperature environments, the optimal design methodology for tapered waveguide units was proposed in the present study. Firstly, the feasibility of the quasi-fundamental shear horizontal (SH0*) wave propagating in the tapered waveguide units was analyzed via numerical simulations, and the transmitting limitations of the non-dispersive SH0* wave were researched. Secondly, several tapered waveguide transducers with varying cross-sections to transmit pure SH0* wave were designed according to the numerical results. Experimental investigations were carried out, and the results were compared with waveguide transducers with a prismatic cross-section. It was found that the tapered waveguide units can transmit non-dispersive shear horizontal waves and suppress the wave attenuation at the same time. The experimental results agreed very well with the numerical simulations. Finally, high-temperature experiments were carried out, and the reliability of thickness measuring by the tapered waveguide transducers was validated. The errors between the measured and the true thicknesses were small. This work paves a solid foundation for the optimal design of tapered waveguide transducers for thickness monitoring of equipment in harsh environments.

## 1. Introduction

There are many pressure vessels and pipes working in harsh environments, e.g., high temperature or corrosive. Online monitoring of the wall thinning caused by erosion or corrosion is very important for the safe operation of such equipment utilizing permanently installed transducers [1,2,3]. However, transducers will suffer from depolarization when they are exposed to high temperature or corrosion for a long time which considerably limits the application for conventional sensory devices in structural health monitoring (SHM) and non-destructive evaluation [4,5]. On the other hand, through investigation it was discovered that waves could propagate in waveguide units, which enables the monitoring of critical mechanical structures working in harsh environments by avoiding the direct immersion of sensors in high temperature regions [6,7]. Many waveguide units of different shapes have been proposed and applied in SHM. Ono et al. [8] designed buffer rods for ultrasonic monitoring at elevated temperatures. In a real engineering application, higher-order modes can also propagate through the rods, and thus dispersion cannot be avoided. However, multiple wave modes and dispersion increased the difficulties of signal processing, so that the measurement accuracy cannot meet the engineering requirements. In order to improve the accuracy of the measurement, several efforts have been made to minimize the adverse effects of dispersion and scattering through the waveguide unit. Jen et al. [9,10] added a cladding layer on the surface of an ultrasonic buffer rod to reduce redundant wave modes. To suppress wave dispersion, Lynnworth et al. [11,12] designed an ultrasonic system composed of hundreds of slender buffer rods with different diameters. Heijnsdijk et al. [13] developed a spiraled sheet waveguide made of a coiled foil, metal or ceramic to transmit non-dispersive wave for flow measurement of a hot fluid in a pipeline. Furthermore, Cawley et al. [14,15,16,17] conducted a series of studies on a uniform rectangular cross-section waveguide unit to transmit the fundamental wave modes in order to excite a single wave mode. Joo et al. [18] proposed a striped waveguide unit for the purpose of transmitting a single A0 mode Lamb waves. Moreover, Young et al. [19] designed waveguide units with a prismatic cross-section and a tapered waveguide device to improve directivity of the interrogating waves. All these different methods can improve the measurement accuracy of the transducers. 

The current study compared all the methods mentioned above; to excite a single wave mode is the best way to improve the measurement accuracy. Moreover, the performance of different wave modes that can be transmitted in the aforementioned waveguide units were studied. It is found that the quasi shear horizontal wave (SH0*) is the most promising wave mode to monitor pressure equipment because of its several beneficial aspects for SHM. An apparent one is the non-dispersive feature which would significantly reduce the difficulties in signal processing and interpretation. Another resides in the fact that the particle motion of the SH0* mode is parallel to the surfaces of the plate without any out-of-plane particle displacement, making it more robust by the presence of surrounding media. 

In order to excite the single non-dispersive SH0* mode, the excitation sources are usually loaded on the thin ends of the long pieces of waveguide units [14,15,16]. In fact, the areas of the thin ends are too small to hold piezoelectric wafers, and it is hard to manufacture such kinds of transducers. Moreover, if the wafers are mounted on the side surface of the waveguide units, the A0* and S0* modes will be excited, accompanying the SH0* wave [16]. However, the pure SH0* wave can be excited when the wafers are installed on the end side of the units [20]. Therefore, in order to excite pure SH0* mode wave, a specially engineered tapered waveguide transducer is proposed in the present research. The thick end is proposed with a bigger cross-sectional area, which provides convenience for the installation of piezoelectric elements. The thin end was designed with a smaller cross-sectional area, which takes the heat dissipation into account. However, there is little theoretical basis for the design of the tapered waveguide transducer to excite pure SH0* mode wave in the current literature. Therefore, the propagation mechanism of the SH0* wave in the tapered waveguide units with a varying cross-section is investigated in Section 2.1., the structural critical parametric values of the tapered waveguide transducer to excite the pure SH0* are studied in Section 2.2. Some tapered waveguide transducers were designed based on the derived theory, and their performances were verified by experiments. Moreover, comparisons between numerical and experimental results were performed in Section 3. High temperature experiments were carried out to validate the reliability of tapered waveguide transducers in Section 4, and the research is concluded in Section 5.

## 2. Propagation Characteristics of SH0* Wave Propagation in Tapered Waveguide Units

**Figure 1:**
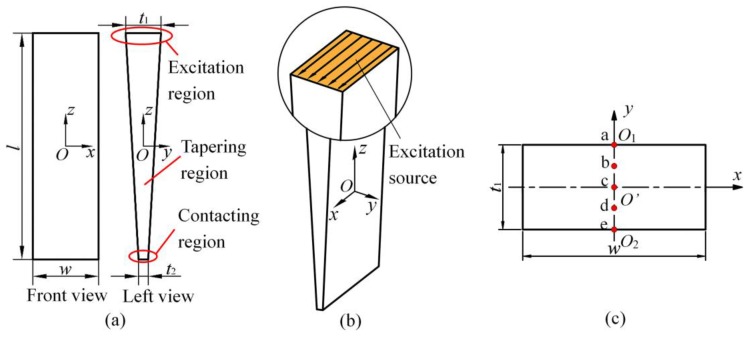
Diagram of the tapering waveguide transducer: (**a**) two-dimensional structure diagram; (**b**) locally zoom-in stereogram; (**c**) top view of the thick end and extracted nodes in the excitation region.

Many analytical and numerical studies on the propagation behavior [21] and scattering characteristics [22] of SH waves have been studied. However, less experimental investigations have been reported mainly due to the difficulties in generating SH waves. Electromagnetic acoustic transducers (EMATs) [19,23] can generate SH waves in tapered waveguide units. Reference [23] reported that the SH0 mode can propagate easily with less reflection when the thickness change is smoother and there is no mode conversion into the SH1 mode. Therefore, we designed our tapered waveguide units with a prismatic cross-section in a gradually changing side length which is similar with the middle part of the structure in Reference [19]. However, the excitation region in Reference [19] is located on the thinnest end, and the excitation region in the current research is located on the thickest end as shown in Figure 1. When we take the excitation source as an object of reference, the gradually changing trend of the waveguide unit is just the opposite. The wave propagates in the way of radiation in Reference [19], but the wave propagates in the way of aggregation in the current research. The wave propagation characteristics in the unit maybe different, because the boundary of the waveguide unit interferes with the wave in a different style. Therefore, we discuss the influence of the structure parameters of the waveguide unit on the wave propagation. 

### 2.1. Feasibility of SH0* Wave Propagation in Tapered Waveguide Units

In this section, the feasibility of SH0* mode propagation in a tapered waveguide with a varying cross-section is analyzed by the finite element method. The two-dimensional structure diagram is shown in Figure 1a. The length, *l*, of the tapered waveguide was 200 mm. The width and the thickness of the thin end was selected according the critical value of the strip waveguide to transmit pure SH0* wave [24]. The width, *w*, of each cross-section was 25 mm. The thickness of each cross-section was uniformly and proportionally reduced from the thick end to the thin end. The thickness of the thick end, *t*_1_, was 5 mm, and the thickness of the thin end, *t*_2_, was 1 mm. The waveguide was made of 316 L of stainless steel with a Young’s modulus of 195 Gpa, a Poisson’s ratio of 0.267, and the density of 7966 kg/m^3^. The structure was discretized using the solid164 element in ANSYS commercial software. The maximum size of the elements was set to 0.2 mm, and the calculation time domain was 200 μs.

An anti-plane shear loading source is applied on the thick end, and the location of the excitation source region is shown in Figure 1b. The excitation signal takes the form a 10-cycle sinusoidal displacement tone burst modulated by a Hanning window at the center frequency of 1 MHz. Echo signals at each node are extracted in the cross-section of the thick end. The extracted nodes are shown in Figure 1c.

**Figure 2:**
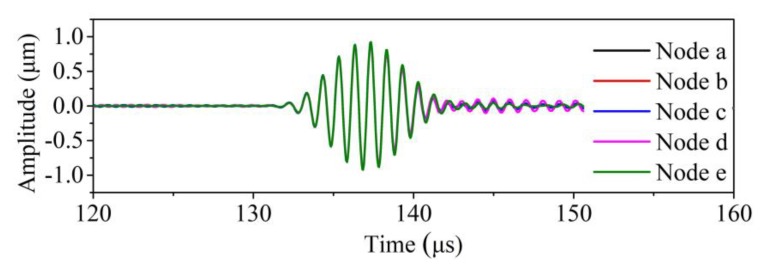
Waveforms in the tapered waveguide unit with varying cross-section.

To enable the analysis of the waveforms in the waveguide units, nodes distributed in line *O*_1_*O*_2_ were utilized, with *O*_1_*O*_2_ being the centerline of the end section along the thickness direction. Typical waveforms of the echo waves were extracted and are presented in Figure 2. It can be seen from the figure that the echo signals demonstrate a remarkable signal-to-noise ratio. Furthermore, the wave comes out to be pure SH0* mode, judged by the wave velocity. This phenomenon confirms that the SH0* wave can propagate in the tapered waveguide structure, when the excitation source is on the thick end.

### 2.2. Limitations of Tapered Waveguide Units for SH0* Mode Propagation

A tapering waveguide unit can be defined by four parameters. The length depends mainly on the temperature of the specimen, the width and thicknesses of the thin end, and the thick end will affect the dispersion of waves [20,24]. In this section, our focus concentrates on the propagation of the SH0* mode wave, while the temperature was not considered in the present research. Therefore, the length, *l*, was selected as a constant, equaling to 150 mm. In addition, the thickness of the thin end was set to 1 mm according to the first cutoff frequency-thickness product [24]. Since there is little theoretical basis for the design of width, *w*, and thickness, *t*_1_, of the thick end in the current literature, the effects of the width, *w*, and the thickness, *t*_1_, will be investigated by finite element simulations.

#### 2.2.1. The Width of the Tapered Waveguide Unit

Based on the simulated results in Section 2, the length of the tapered waveguide unit was chosen as 150 mm, and the thicknesses of the thick and thin ends were 5 mm and 1 mm, respectively. The width, *w*, was parameterized from 7 mm to 25 mm at an interval of 1 mm. The excitation source was loaded on the thick end, and the simulation conditions were the same as those in Section 2. 

**Figure 3:**
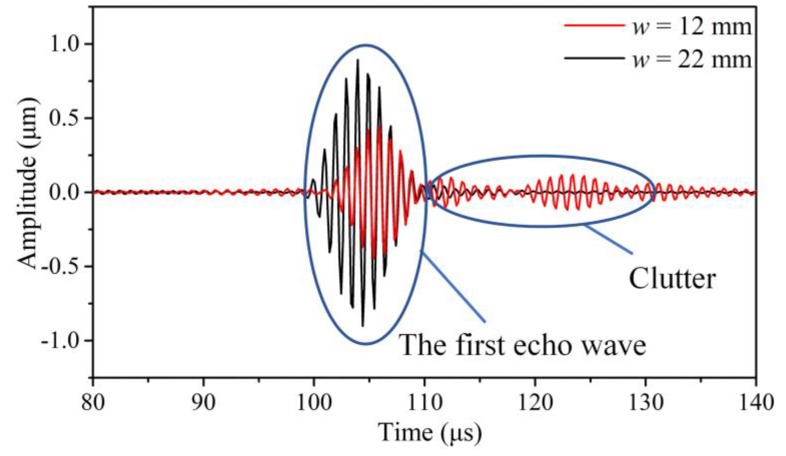
Comparative waveforms when *w* is 12 mm and 22 mm.

The echo signals were extracted from the center point of the thin end of the tapered waveguide unit. Figure 3 shows the comparative waveforms in the waveguide unit, the width, *w*, of which was 12 mm and 22 mm. It can be seen that waves in the tapered waveguide unit dispersed when the width, *w*, was 12 mm. There are several waveforms in the limited time region, and the velocity of the main waveform came out to be slower than the group velocity of the SH0* wave. On the other hand, the wave propagating in the tapered waveguide unit was pure when the width, *w*, was 22 mm. The signal amplitude of the excitation waveform becomes much higher and possesses a much better signal-to-noise ratio. In addition, the wave velocity was approximately equal to the group velocity of the SH0* wave in a plate.

**Figure 4:**
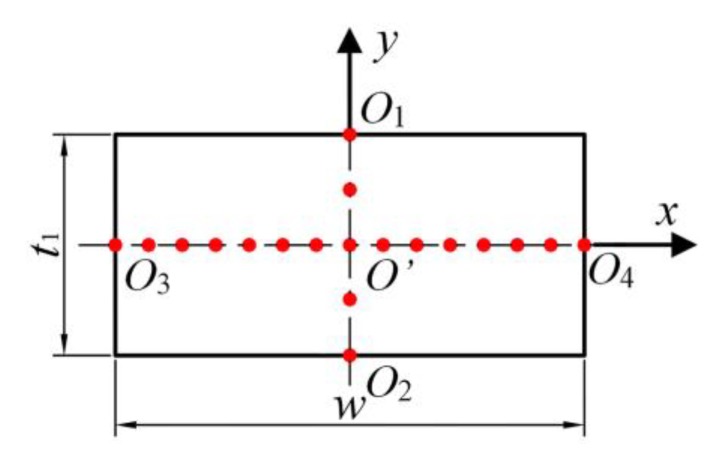
The representative nodes in the excitation region.

In order to identify which kind of waves could be excited in the tapered waveguide unit, the wave structures were analyzed, and the echo signals of representative nodes were extracted. The nodes lie in *O*_1_*O*_2_ and *O*_3_*O*_4_ lines as shown in Figure 4. Both *O*_1_*O*_2_ and *O*_3_*O*_4_ were orthogonal centerlines of the thick end section. The line *O*_1_*O*_2_ resided along the thickness direction. The line *O*_3_*O*_4_ was along the width direction.

**Figure 5:**
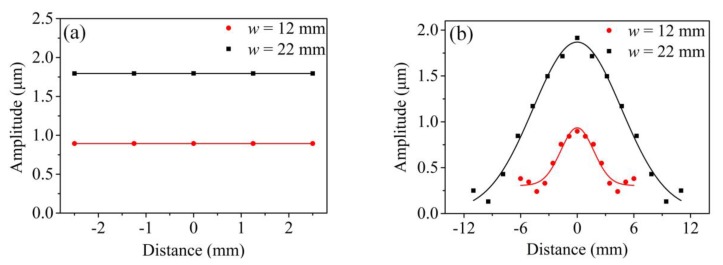
Displacement amplitude of nodes on the thick end of waveguide unit: (**a**) thickness direction; (**b**) width direction.

The displacement amplitudes of echo signals of each node are compared in Figure 5. Echo signals of nodes in the thickness direction are shown in Figure 5a and those in the width direction are presented in Figure 5b. It can be seen from Figure 5a that the displacement amplitude of each node in the thickness direction was equal, independent of the width condition. This phenomenon indicates that the wave structure along the thickness direction was alike the SH0* wave in the uniformly thick waveguide. Figure 5b shows the change of displacement amplitude in the width direction of the waveguide unit. It was noticed that the displacement amplitudes of waves decreased when the nodes were far away from the midpoint of line *O*_3_*O*_4_. Moreover, the displacement amplitudes on the edge will asymptotically approach zero when the waveguide unit is wide enough. That is to say, the wave energy mainly centralizes in the middle of the waveguide unit. This phenomenon also indicates that the wave was alike the SH0* wave in the uniformly thick waveguide.

**Figure 6:**
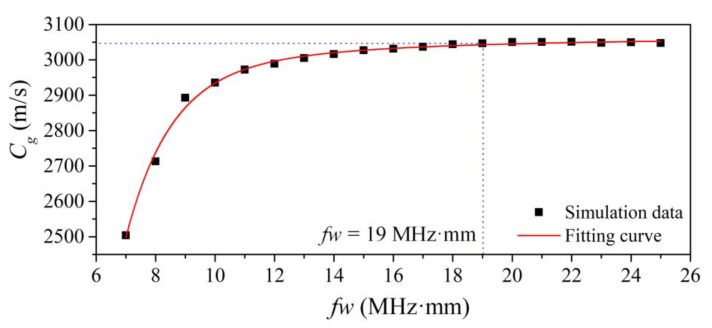
Group velocity versus frequency–width product.

After examining the wave structure in the waveguide unit, the group velocities were calculated according to the wave propagation distance and the time of flight of the echo signals. The curve of group velocities versus the frequency–width product *fw* is shown in Figure 6. It was noticed that the group velocities in the waveguide unit were related to *fw*. This curve verifies that the waves in tapered waveguide unit depended on the *fw*. The wave velocity was slow, because the wave will disperse and become distorted at low *fw.* The transition from highly dispersive to non-dispersive was virtually complete at a frequency–width product of 19 MHz·mm. When *fw* > 19 MHz·mm, the group velocity approximated that of the fundamental shear horizontal wave in a plate. This means high frequency–width products allow non-dispersive wave propagation without signal distortion. The SH0* mode phase velocity was constant and equal to the bulk shear velocity.

According to the above analyzed wave characteristics, the tapered waveguide unit can transmit non-dispersive SH0* when the product of frequency and width is greater than 19 MHz·mm. 

#### 2.2.2. The Thickness of the Thick End t_1_

According to the conclusion drawn in Section 2.2.1., the width can be chosen as 20 mm when the simulated frequency is 1 MHz with *l* being 150 mm and *t*_2_ being 1 mm. The thickness *t*_1_ of the thick end was changed from 1 mm to 13 mm at an interval of 1 mm. The echo waves were extracted at the central point *O’* at the thick end of the waveguide unit.

**Figure 7:**
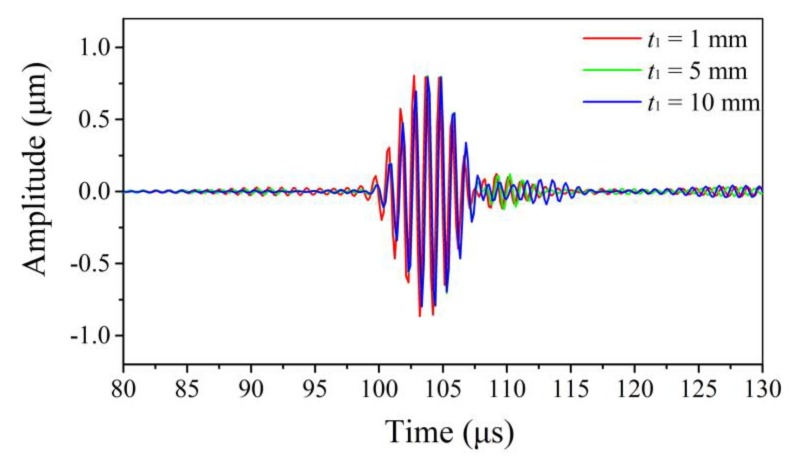
Waves in time domain for *t*_1_ = 1 mm, 5 mm, and 10 mm.

The waveforms transmitted in the waveguide units with different *t*_1_ (i.e., 1 mm, 5 mm, and 10 mm) are shown in Figure 7. It was noticed that the waveforms shared great resemblance: all of them possessed high signal-to-noise ratios. As a matter of fact, all the simulated waveforms looked like each other when *t*_1_ of the waveguide unit increased from 1 mm to 13 mm, showing that the waves did not disperse. Therefore, it can be concluded that the thickness *t*_1_ of the thick end of the waveguide unit had little influence on the non-dispersive feature of shear horizontal guided waves.

Therefore, we can conclude that the SH0* wave can propagate in the tapered waveguide structure whether the excitation source is on the thick end or the thin end. The width and thicknesses of the thin end are the dominant parameters which will affect the dispersion of waves, and wave dispersion is independent of the thickness of the thick end. When the frequency–thick product of the thin end is less than the first cutoff frequency–thickness product and the frequency–width product is bigger than the critical value, this allows non-dispersion of the SH0* wave propagation without signal distortion. The critical value of the tapered waveguide unit is approximately equal to that of the uniformly thick strip waveguide unit. Moreover, wave structure in the tapered waveguide is alike the SH0* wave in the uniformly thick waveguide. 

## 3. Experimental Verification of SH0* Wave Propagation in Tapered Waveguide Units

**Figure 8:**
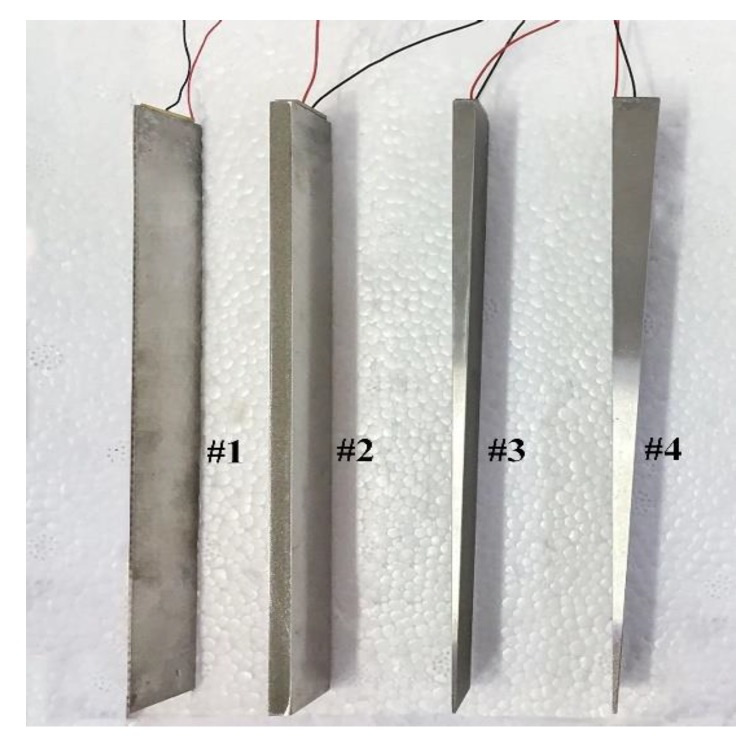
Picture of the tapered waveguide transducers: the waveguide units of the #1 and #2 transducers are with prismatic cross-sections and #3 and #4 are tapered with varying cross-sections.

**Figure 9:**
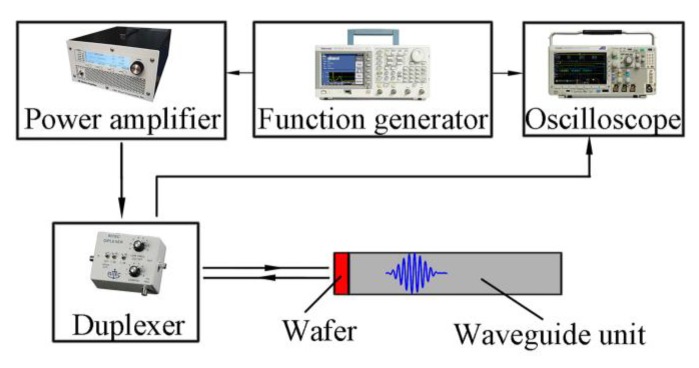
Diagram of experimental system.

In this section, the propagation of the SH0* wave will be investigated in tapered waveguide units by experiments. The experimental system includes oscilloscope MDO 3012 (Tektronix, INC., Beaverton, OR, USA), function generator AFG 3021C (Tektronix, INC., Beaverton, OR, USA), power amplifier AG1006 (T&C Power Conversion, INC., Rochester, NY, USA), diplexer, piezoelectric wafers, and waveguide units. The material of the piezoelectric wafers was PZT-5H, while that of the waveguide units is 316L. The piezoelectric wafer was attached to the top end of the waveguide units using epoxy resin as shown in Figure 8. Number #1 and #2 were waveguide units with prismatic cross-sections and #3 and #4 were tapered waveguide units with varying cross-sections, their detailed structural dimensions are listed in Table 1. The structure sizes of #1 and #2 units were designed according to the critical values in Reference [24]. The structure sizes of the #3 and #4 units were designed according the limitations derived in the above sections. The surface areas of the wafers equate to the thick end areas. During testing, the ten-cycle tone bursts modulated by a Hanning window are generated. The signal gain is kept constant for all the experiments. The excited guided wave propagates along the waveguide units and gets reflected from the bottom of the units, and then picked up by the piezo-wafers. The experimental system is illustrated in Figure 9.

**Figure 10:**
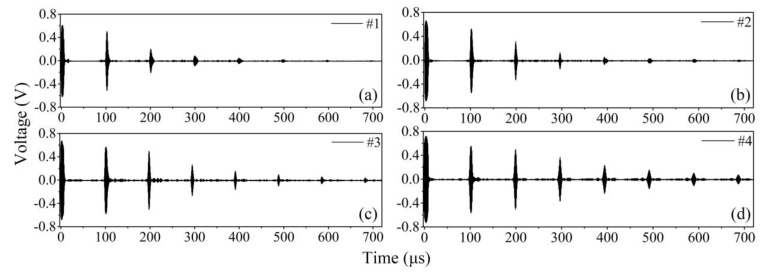
Waveforms in waveguide units with equal cross-sections and varying cross-sections: (**a**) the unit with prismatic cross-section #1; (**b**) the unit with prismatic cross-section #2; (**c**) the tapered unit with varying cross-section #3; (**d**) the tapered unit with varying cross-section #4.

The experimental waveforms in temporal domain are presented in Figure 10. Figure 10a,b demonstrates the waveforms in the waveguide units with prismatic cross-sections, and Figure 10c,d shows the waveforms in the tapered waveguide units with varying cross-sections. All the wave signals are clear and non-dispersive. They are SH0* wave judged by wave velocity. Therefore, it can be concluded that the SH0* wave can be effectively transmitted in these units. Comparing Figure 10a,b, it can be seen that the echo signal energy propagating through the waveguide unit with prismatic cross-sections amplified when the thickness of the waveguide unit increases from 1 mm to 5 mm which indicates that the energy of the echo signal amplified with the increment of the excitation source area, and the wave dispersion was independent of the thickness of the thick end. Comparing Figure 10b,c, it can be seen that the echo energy of SH0* wave in the tapered waveguide of unit #3 was larger than that in waveguide unit #2 with a prismatic cross-section. It was shown that the tapered structure can effectively reduce the attenuation of wave propagation in the units. This is caused by the wave transmission. The transmission area of the waveguide unit #2 was bigger than that of the tapered waveguide unit #3. The transmitting energy will be dissipated in the air. That is to say, the tapered waveguide unit, in which waves propagate in the way of aggregation, was better than that of the waveguide unit with a prismatic cross-section to transmit the SH0* wave. It can be noticed from Figure 10c,d that, for the tapered structure, when the thickness of the unit increased from 5 mm to 10 mm, the energy of the echo signal in the waveguide unit amplified, and the wave could go back and forth many times which indicated that the wave energy amplified with the increase in the excitation source. Therefore, it can be concluded that the tapered waveguide unit with varying cross-sections can effectively transmit non-dispersive SH0* waves and reduce the attenuation of waves in the propagation process. In other words, the tapered waveguide unit is preferred more for practical engineering applications.

## 4. High Temperature Experimental Validation of Tapered Waveguide Transducers

**Figure 11:**
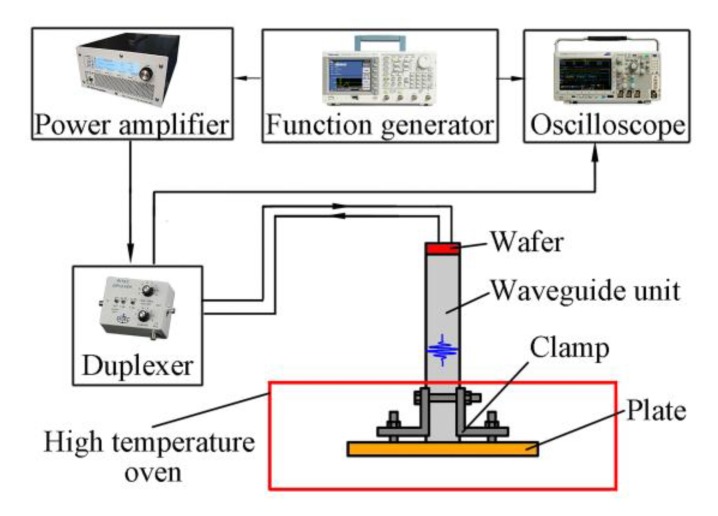
Diagram of the experimental system.

In this section, the tapered waveguide transducers will be used for thickness measurement at high temperature to verify their reliability. In high temperature experiments, the equipment system is almost the same as in Section 3, except for employing a 316L stainless steel plate (150 mm × 150 mm × 10 mm) as the test target and a high temperature oven to induce the harsh environment. The diagram of the experimental system is shown in Figure 11. Specific dimensions of the bars and wafers are listed in Table 2. The length of the wafer is *l*_0_; *w*_0_ stands for the width; *t*_0_ represents the thickness_._ The wafers are mounted on the top end of the waveguide units and replace the excitation resource in Figure 1b. Differently, a 1 mm margin was reserved at the edge of the width to avoid the influence of the clamping tool on wave propagation. The tapered waveguide transducers are installed on the test plate by the thin end of the transducers using the specially designed clamping tool. The thick end of the transducers reached outside of the furnace through a hole at the top of furnace, creating a clearance distance of about 250 mm between the furnace and the wafer location. The high temperature oven was heated to 350 °C. The five-cycle Hanning window modulated tone bursts were generated at the center frequency of 1 MHz, and the sensing signals were acquired after holding the temperature for about one hour. The products of frequency and width of the waveguide transducers were equal to 20 MHz·mm, which was bigger than the critical values derived in Section 3.

**Figure 12:**
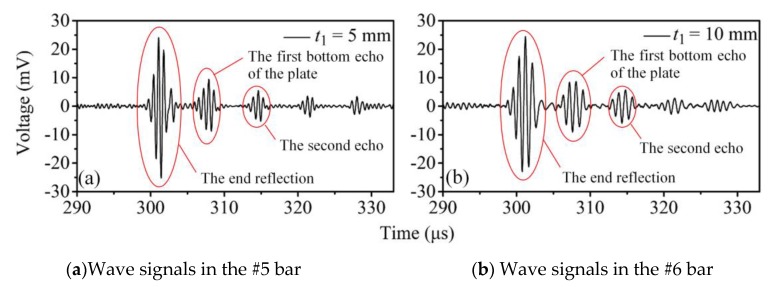
The waveforms in high temperature experiments: (**a**) *t*_1_ = 5 mm; (**b**) *t*_1_ = 10 mm.

The acquired waveforms are shown in Figure 12. The wave packets in the figures are, consecutively, the end reflection of the waveguide units, the first bottom echo of the plate, the second echo, the third echo, etc. The end reflection of the waveguide units and the first bottom echo of the plate are marked out. It can be observed that the waveforms were clear with high signal-to-noise ratios. The energy of the echo signal was strong enough, and the wave could go back and forth many times. The group velocity approximates that of the fundamental shear horizontal wave in a plate. Moreover, the waves excited in both waveguide bars with different structural sizes verify that the dispersion characteristics of wave signals excited by different transducers were independent of the thickness of the thick end. These results are consistent with that in Section 3. 

Moreover, according to the time-of-flight *t* of each wave-packet peak in Figure 12a,b as well as the shear wave speed *v* = 2900 m/s, the thickness of the specimen can be calculated using the formula *s* = *vt*/2. The measured thickness values were 9.94 mm and 9.99 mm, respectively. The thickness of the tested plate was 9.96 mm which was measured by the high–precision thickness gauge EPOCH650 (Olympus NDT, Inc., Waltham, MA, USA). Thus, the measurement errors of the high temperature experiment can be solved and turned out to be less than 3% which satisfies most of practical engineering requirements. 

The measured thickness results were considerably reliable which verifies that the design methodology of the proposed tapered waveguide transducers derived in the present research were reliable. 

## 5. Conclusions

The optimal design methodology of specially engineered waveguide transducers for the thickness monitoring of pressure equipment was presented. Firstly, the propagation mechanism of SH0* wave in the tapered waveguide unit with varying cross-sections was analyzed by numerical simulations. It was found that the SH0* wave can propagate in the tapered waveguide unit just like that in strips. Secondly, the structural limitations of the tapered waveguide unit with varying cross-sections to propagate the non-dispersive SH0* wave were studied. We have found that the width and thicknesses of the thin end were the dominant parameters that will affect the dispersion of waves; wave dispersion is independent of the thickness of the thick end. When the frequency–width product was bigger than the critical value and the frequency–thickness product was less than the first cutoff value, the higher wave modes were avoided and the non-dispersive SH0* wave could propagate without being distorted. Finally, typical waveguide units were designed according to the derived design criterion and experiments were carried out. The experimental results illustrated that the tapered waveguide unit with varying cross-sections could effectively transmit non-dispersive SH0* waves and reduce the attenuation of the SH0* wave at the same time. Moreover, the thickness of the specimen plate exposed to the high temperature environment was measured using the different tapering waveguide transducers. The thickness testing results were reliable, and the measuring errors satisfy most engineering requirements. Therefore, a conclusion can be drawn that the design methodology of tapered waveguide transducer is suitable for practical engineering applications. Field applications of the tapered waveguide transducer will be a next-step endeavor to verify the practical monitoring reliability of wall thinning of the pressure equipment in harsh environment.

## Figures and Tables

**Table 1:** The structural dimensions of the waveguide units.

Number	*t*_1_/mm	*t*_2_/mm	*w*/mm	*l*/mm
#1	1	1	20	150
#2	5	5	20	150
#3	5	1	20	150
#4	10	1	20	150

**Table 2:** The detailed structural dimensions of bars and wafers.

Number	The Tapered Waveguide Bar				The Piezoelectric Wafer		
	*l*/mm	*w*/mm	*t*_1_/mm	*t*_2_/mm	*l*_0_/mm	*w*_0_/mm	*t*_0_/mm
#5	450	20	5	1	18	5	0.5
#6	450	20	10	1	18	10	0.5
